# First Molecular Detection of *Polychromophilus* Parasites in Brazilian Bat Species

**DOI:** 10.3390/microorganisms9061240

**Published:** 2021-06-07

**Authors:** Guilherme Augusto Minozzo, Bruno da Silva Mathias, Irina Nastassja Riediger, Lilian de Oliveira Guimarães, Carolina Clares dos Anjos, Eliana Ferreira Monteiro, Andrea Pires dos Santos, Alexander Welker Biondo, Karin Kirchgatter

**Affiliations:** 1 Laboratório Central de Saúde Pública do Paraná, São José dos Pinhais 83060-500, PR, Brazil; guilhermeaminozzo@gmail.com (G.A.M.); irinariediger@sesa.pr.gov.br (I.N.R.); 2 Programa de Pós-Graduação em Medicina Tropical, Instituto de Medicina Tropical, Faculdade de Medicina, Universidade de São Paulo, São Paulo 05403-000, SP, Brazil; brunomathiasbio@gmail.com (B.d.S.M.); carolinaclares@gmail.com (C.C.d.A.); elianafmonteiro@usp.br (E.F.M.); 3 Laboratório de Bioquímica e Biologia Molecular, Superintendência de Controle de Endemias, São Paulo 01027-000b, SP, Brazil; lilianguima@gmail.com; 4 Department of Comparative Pathobiology, Purdue University, West Lafayette, IN 47907, USA; santos1@purdue.edu; 5 Departamento de Medicina Veterinária, Universidade Federal do Paraná, Curitiba 80035-050, PR, Brazil; abiondo@ufpr.br

**Keywords:** *Polychromophilus*, bats, phylogeny, Brazil

## Abstract

Blood parasites of the Haemosporida order, such as the *Plasmodium* spp. responsible for malaria, have become the focus of many studies in evolutionary biology. However, there is a lack of molecular investigation of haemosporidian parasites of wildlife, such as the genus *Polychromophilus.* Species of this neglected genus exclusively have been described in bats, mainly in Europe, Asia, and Africa, but little is known about its presence and genetic diversity on the American continent. Here, we investigated 406 bats from sites inserted in remnant fragments of the Atlantic Forest and Cerrado biomes and urbanized areas from southern Brazil for the presence of *Polychromophilus* species by PCR of the mitochondrial cytochrome b encoding gene. A total of 1.2% of bats was positive for *Polychromophilus*, providing the first molecular information of these parasites in *Myotis riparius* and *Eptesicus diminutus*, common vespertilionid bats widely distributed in different Brazilian biomes, and *Myotis ruber*, an endangered species. A Bayesian analysis was conducted to reconstruct the phylogenetic relationships between *Polychromophilus* recovered from Brazilian bats and those identified elsewhere. Sequences of Brazilian *Polychromophilus* lineages were placed with *P. murinus* and in a clade distinct from *P. melanipherus*, mainly restricted to bats in the family Vespertilionidae. However, the sequences were split into two minor clades, according to the genus of hosts, indicating that *P. murinus* and a distinct species may be circulating in Brazil. Morphological observations combined with additional molecular studies are needed to conclude and describe these *Polychromophilus* species.

## 1. Introduction

The phylum Apicomplexa forms one of the most diverse groups of unicellular protists with a wide environmental distribution. They are classified as mandatory intracellular parasites and they have mobile invasive stages. They are characterized by the presence of an evolutionarily unique structure called the apical complex, used to adhere and invade host cells. Many of the species that are part of this group are considered pathogens in humans and other vertebrates. All animal species are believed to host at least one species of apicomplexan parasites [1,2,3]. Apicomplexa are divided into two orders: Eucoccidiorida (coccidian parasites) and Haemosporida (haemosporidian parasites). Haemosporida are organized into four families: Garniidae, Haemoproteidae, Leucocytozoidae, and Plasmodiidae, which include malaria parasites that infect various vertebrates and invertebrate hosts [4].

The hosts of the order Chiroptera have the greatest diversity of haemosporidian parasites among mammals, including nine genera. In addition to the well-known genera (*Plasmodium* and *Hepatocystis*), seven genera exclusively infect chiropterans: *Polychromophilus*, *Nycteria*, *Bioccala*, *Biguetiella*, *Dionisia*, *Johnsprentia*, and *Sprattiella* [5,6], clearly highlighting this group of mammals as a vital tool in the taxonomic, systematic, and evolutionary study of haemosporidians in mammals. Although *Bioccala* was elevated to a genus in 1984 [7], many studies, as well as this work, still use it as a subgenus of *Polychromophilus,* since its species present similar morphological characteristics and its genetics have not been studied [8].

The genus *Polychromophilus* has been found in insectivorous bats in tropical and temperate regions [9,10,11,12]. Only five species of *Polychromophilus* are known. Although they can be distinguished by slight differences in ultrastructure, they are classified mainly based on the type of host [13]. Of the five species of *Polychromophilus* described, *Polychromophilus* (*Polychromophilus*) *melanipherus* and *Polychromophilus* (*Bioccala*) *murinus* are mainly linked to two bat families: Miniopteridae and Vespertilionidae, respectively [14]. However, occasionally, *P. melanipherus* has been reported in Hipposideridae and Vespertilionidae and *P. murinus* in Rhinolophidae, Hipposideridae, and Miniopteridae [6]. In addition, the species *P.* (*P*.) *corradetti* and *P*. (*P*.) *adami* have been described in bats from the African region: *Miniopterus inflatus* in Gabon and *Miniopterus minor* in the Republic of Congo [13].

Recent studies have demonstrated a greater concentration of molecular studies aimed at African and European bats, e.g., [8,15,16,17]. In contrast, our knowledge about haemosporidian parasites of Brazilian bats is still restricted to morphological investigations, such as the case of *Polychromophilus* (*Bioccala*) *deanei* found in *Myotis nigricans* (Vespertilionidae). *Myotis nigricans* is an evening bat from Brazil, and is the first chiropteran host in which this group of parasites was found in the New World [18,19]. Nevertheless, no molecular data is available for this parasite in Brazil, and the only sequence of *Polychromophilus* sp. of bats from the American continent is from *Myotis nigricans*, from the Vespertilionidae family, found in Panama [20].

## 2. Materials and Methods

### 2.1. Sampling

**Figure 1:**
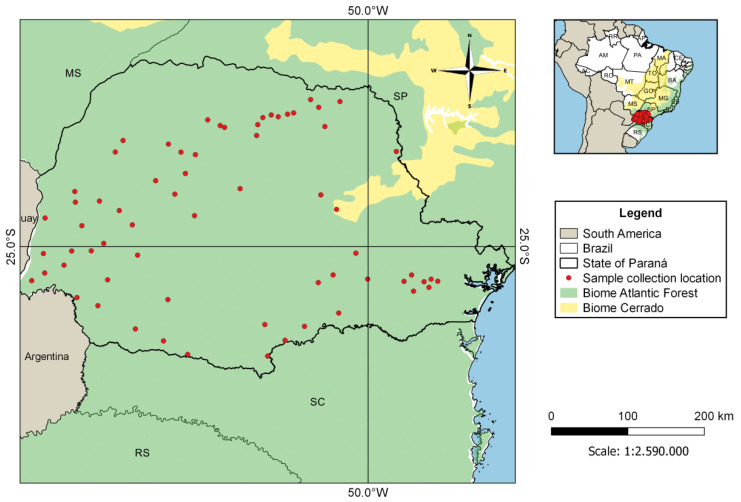
Location of municipalities in the State of Paraná, Brazil, where bat samples were collected.

Brain tissue samples of bats with no identified species (*n* = 406) were acquired from the Parana State Reference Laboratory (LACEN) program for monitoring rabies virus circulation. They were collected between September 2019 and August 2020 in 67 different municipalities in the State of Paraná, most of them inserted in remnant fragments of Atlantic Forest and Cerrado biomes, as well as in urbanized areas (Figure 1).

All tissue samples and bats were collected and handled under appropriate authorizations by the Brazilian government. The project was approved by the Ethics in Use of Animals Committee, CEUA/SESA, of the Centro de Produção e Pesquisa de Imunobiológicos—CPPI/PR (approval number 01/2019 and date of approval 3 March 2020).

### 2.2. Polychromophilus Detection

The extraction of total nucleic acid (DNA and RNA) from collected samples was performed using the BioGene Extraction kit (K204-4, Bioclin, Belo Horizonte, MG, Brazil), following the manufacturer’s instructions.

A fragment of ~1.1 kb (approximately 92% of the gene) from the mitochondrial cytochrome b gene (*cytb*) was amplified using a nested polymerase chain reaction (PCR), taking standard precautions to prevent cross-contamination of samples. The PCR reactions were conducted as previously described [21] using primers DW2 and DW4 and 5 uL of genomic DNA in the first reaction, and 1 uL aliquot of this product was used as a template for a nested reaction with primers DW1 and DW6.

PCR products were sequenced using BigDye^®^ Terminator v3.1 Cycle Sequencing Kit in ABI PRISM^®^ 3500 Genetic Analyzer (Applied Biosystems, Carlsbad, CA, USA) using nested PCR primers. The *cytb* sequences were obtained and aligned with the sequences available at the GenBank^®^ database.

The phylogenetic relationship among reported parasites was inferred using partial *cytb* gene sequences (1116 bp). GenBank^®^ accessions of the used sequences are shown in the phylogenetic trees. The phylogenetic reconstruction was performed using the Bayesian inference method implemented in MrBayes v3.2.0 [22]. Bayesian inference was executed with two Markov Chain Monte Carlo searches of 3 million generations, with each sampling 1 of 300 trees. After a burn-in of 25%, the remaining 15,002 trees were used to calculate the 50% majority-rule consensus tree. The phylogeny was visualized using FigTree version 1.4.0 [23].

### 2.3. Host Species Identification

The positive samples were processed using a PCR protocol that amplifies host DNA with primers L14841 and H15149 that were designed to amplify fragments with ~390 bp of the mitochondrial *cytb* gene from a wide range of animals, including mammals, birds, amphibians, reptiles, and fish [24]. Amplified fragments were sequenced directly using the corresponding flanking primers. Obtained sequences were compared to other sequences deposited in the GenBank^®^ database (www.ncbi.nlm.nih.gov/blast/Blast.cgi accessed on 19 March 2021). The best close match (BCM) algorithm was used to identify the best barcode matches of a query, and the species name of that barcode was assigned to the query if the barcode was sufficiently similar [25]. Positive identification and host species assignment were made when sequences presented a match of >97%.

Alternatively, for some specimens, a fragment with ~650 bp from the mitochondrial cytochrome c oxidase (*coi*) gene was amplified by two methods: (i) using the primers VF1_t1 (5′-TGT AAA ACG ACG GCC AGT TCT CAA CCA ACC ACA AAG ACA TTG G-3′) [26] and VR1_t1 (5′-AGG AAA CAG CTA TGA CTA GAC TTC TGG GTG GCC AAA GAA TCA-3′) [27] with PCR conditions and cycling from Kumar et al. [28], and (ii) using the universal primers LCO 1490 and HCO 2198 [29] and PCR protocol based on Ruiz et al. [30].

## 3. Results

**Figure 2:**
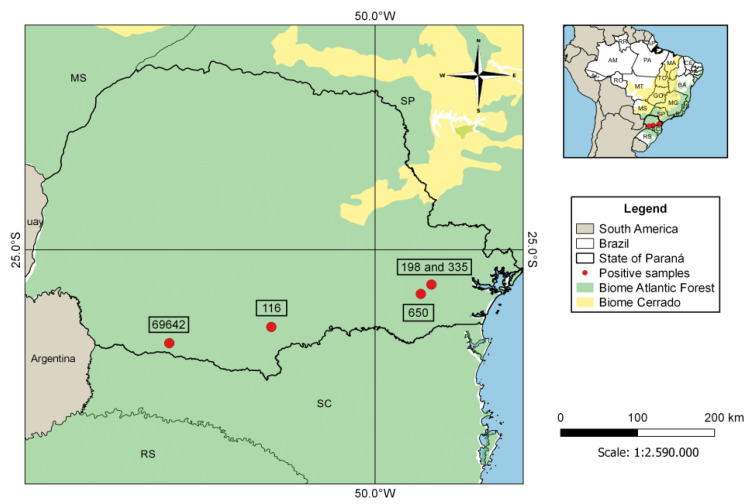
Distribution of the positive samples of *Polychromophilus* sp. isolates from Paraná state, Brazil.

This study detected five samples that were positive for *Polychromophilus* sp. (sample IDs: 116, 198, 335, 650, and 69642), confirming the presence of parasites of this genus in Brazilian bats. The percentage of positives was 1.2% (5/406) of the number of samples analyzed. Accordingly, the sequences of *cytb* and *coi* genes from the positive host samples were from *Myotis ruber* (116), *Myotis riparius* (198, 335, and 69642), and *Eptesicus diminutus* (650), all bats belonging to the Vespertilionidae family, collected in four municipalities in the State of Paraná (Araucaria, Cruz Machado, Curitiba, and Pato Branco) (Figure 2). The two samples obtained in Curitiba city were probably from an urban area since Curitiba is the most populous municipality of Paraná state and the eighth in the country.

The nucleic acid polymorphism in mitochondrial *cytb* sequences (1116 bp) of *Polychromophilus* sp. isolates from Brazil compared to the best match sequence from GenBank^®^ (#LN483038 of *Myotis nigricans* from Panama with 595 bp) is shown in Table 1. Thirteen sites were polymorphic among Brazilian sequences (Table 1). The Panamanian sequence, the only available one obtained from bats from the American continent, showed two nucleic acid substitutions found only in this isolate (gray columns) (Table 1).

The sequence obtained from bat 650 was the most divergent, with 98–99% of identity with the others (with 11 or 12 nucleic acid substitutions) (Table 2). The Panamanian sequence presented two to eight nucleic acid substitutions compared to Brazilian sequences (98–99% of identity) (Table 2).

**Figure 3:**
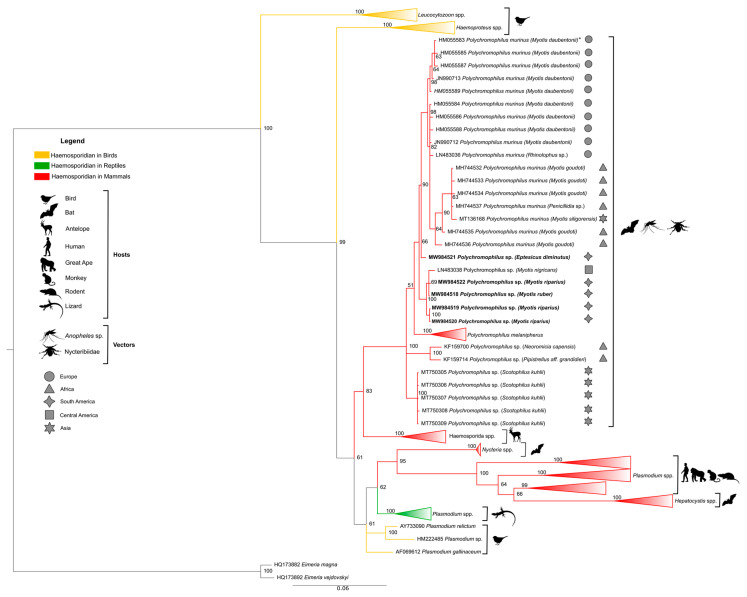
Bayesian phylogeny based on the mitochondrial cytochrome b gene (*cytb*) from *Polychromophilus* spp. of the sequences identified in the present study (1116 bp) and reference sequences listed in Table A1 and Table A2 in Appendix A. * Sequence HM055583 has also been reported in *P. murinus* from *Eptesicus serotinus*, *Nyctalus noctule*, and *Myotis myotis* (Table A2, Appendix A). *Eimeria* spp. were used as an external group. The support values of the nodes (in percentage) indicate posterior probabilities. The red branches highlight the haemosporidian sequences found in mammals. The yellow branches highlight the haemosporidian sequences found in birds. The green branches highlight the haemosporidian sequences found in reptiles. The sequences found in the present study are highlighted in bold. The remaining reference sequences are collapsed to highlight the branch of the *Polychromophilus* genus.

The phylogenetic tree in Figure 3 was generated with reference sequences found in the Genbank^®^ database, covering different haemosporidian genera obtained from different hosts (Table A1, Appendix A). The *Polychromophilus* sequences found in this study and all sequences of the genus available in the Genbank^®^ database (Table A2, Appendix A) were included. The clade of the genus *Polychromophilus* is shown in evidence, and the remaining haemosporidian from other genera were collapsed.

Phylogenetic analysis based on *cytb* did not produce conflict in any of the main nodes. All the main genera and subgenera were recovered and represented in the phylogenetic tree by separate monophyletic clades. The results show the existence of four clades within the Haemosporida order analyzed here. Phylogeny also showed *Polychromophilus* as a sister clade of a group that contains *Plasmodium* species of ungulates, but with a distant relationship between *Plasmodium* and *Hepatocystis* from other mammals, such as primates and rodents.

All *Polychromophilus* sequences from bats of different parts of the world were grouped into a monophyletic clade (posterior probability of 100) composed of four subclades, with all *Polychromophilus* found in Brazilian bats segregated in only one of them. The first distinct subclade comprised all sequences of *P. melanipherus* from *Miniopterus* bat hosts, and the second subclade exclusively included sequences of *Polychromophilus* from vespertilionids (including Brazilian ones), confirming a clear separation of parasites from miniopterid and vespertilionid hosts. The other subclade that was separated contained the *Polychromophilus* sequences from *Scotophilus kuhlii* from Thailand (MT750305-MT750309). Two samples of parasites of *Pipistrellus* aff. *grandidieri* and *Laephotis capensis* from Guinea (KF159700 and KF159714) formed a separate group.

The subclade of *Polychromophilus* from vespertilionids was divided into two branches: one contained sequences of *P. murinus* from bats in Europe (Switzerland, Bulgaria), Madagascar, and Thailand, and a sequence of *Eptesicus diminutus* (650) from Brazil, and the other clade with *M. nigricans* from Panama and all the other Brazilian sequences isolated from the *Myotis* species.

## 4. Discussion

Based on the results presented herein, although the total number of bat families tested is unknown, *Polychromophilus* infection in Brazilian bats appears to be limited to just one family (Vespertilionidae). This finding is in accordance with the only previous report of *Polychromophilus* from Brazil, described as *P. deanei*, found in *Myotis nigricans*, also a Vespertilionidae bat [18,19].

According to one study, Paraná state has poor fauna regarding the number of bat species, with only 53 species from five families recorded [31]. The Phyllostomidae family has the highest species richness (25; 47% of the total), followed by Molossidae (13; 24%), Vespertilionidae (12; 22%), Noctilionidae (2; 4%), and Emballonuridae (1; 2.5%) [31]. Miretzki also showed the occurrence of only 55% of the species of the Atlantic Forest biome and the relative predominance of vespertilionids and molossids over phyllostomids. Herein, we analyzed samples obtained from much of the state’s area, with great sampling opportunities for other families. However, we were unable to find *Polychromophilus* in bat species that were not vespertilionids, suggesting that this parasite may be restricted to this group of bats in Brazil.

Regarding the frequency, we found the lowest positivity rate reported to date, although the total number of samples analyzed herein is one of the highest among published studies (Table 3). This could be related to the sample type analyzed in this study. This was the first time that *Polychromophilus* DNA was obtained from brain tissue, probably from parasites in the blood vessels that irrigate the organ. Thus, the direct comparison of the prevalence data with published studies that used blood samples is impaired.

Three different Brazilian bats species were found to be positive for *Polychromophilus* sp.: two *Myotis* species (*M. ruber* and *M. riparius*) and one species from the *Eptesicus* genus (*E. diminutus*). There are reports of *Myotis* species infections in Africa (*M. tricolor* in Kenya and *M. goudoti* in Madagascar) [17,36,37], Europe (*M. daubentonii* and *M. myotis* in Switzerland) [38], and Asia (*M. siligorensis* in Thailand) [40]. However, the only record of *Polychromophilus* infection in *Eptesicus* comes from Europe (*E. serotinus* in Switzerland) [38].

*Myotis riparius* is present in Honduras, Uruguay, Bolivia, Argentina, Paraguay, Trinidad, and Brazil [41], including the state of Paraná [31,42,43]. *Myotis ruber* is an endangered species under the category of “vulnerable” according to the Brazilian Institute of Environment and Renewable Natural Resources—IBAMA [44], and under the category of “near threatened” at a global level according to IUCN [45]. It is distributed across Argentina, Uruguay, Paraguay [40,46,47,48], and southeastern Brazil, including Paraná [49].

It is important to note that in our molecular identification of the host species using *cytb* and sequence comparisons, *Eptesicus furinalis* was the species with the best close match with the sequence obtained from bat 650. However, the percentage of identity was low (89%) compared to sequences available in the GenBank^®^ database, making it impossible to identify the species. Thus, alternatively, we used the *coi* gene and the BOLD database (https://www.boldsystems.org/ (accessed on 31 March 2021), finding 98% of identity with an *Eptesicus diminutus* sequence, a reliable value for the species identification using the BCM method. *Eptesicus diminutus* has a distribution in the north and east regions of Paraná state [31]. It is from the Vespertilionidae family, and it is absent from the GenBank^®^ database, which explains the first finding. Thus, we considered specimen 650 to be *Eptesicus diminutus*.

Our phylogenetic analysis showed a strongly defined clade represented by *Plasmodium* infecting rodents and primate hosts, which also included *Hepatocystis* isolated from bats. Similar data were obtained by other authors [38,50]. *Haemoproteus* and *Leucocytozoon* species were grouped separately in individual clades, as previously shown [51,52].

Regarding *Polychromophilus* sequences, a similar topology in the phylogenetic tree was obtained by Chumnandee et al. [39], where they grouped into a monophyletic clade with a clear separation of parasites from miniopterid and vespertilionid hosts. Four Brazilian sequences (GenBank^®^ MW984519, MW984520, MW984522 from *Polychromophilus* sp. isolated of *Myotis riparius*, and MW984518 from *Polychromophilus* sp. isolated of *Myotis ruber*) were positioned close to the sequence of *Polychromophilus* sp. of bats of the species *Myotis nigricans,* Vespertilionidae family, from the Latin American region (Panama) (GenBank^®^ #LN483038) [20]. One Brazilian sequence (GenBank^®^ #MW984521, from *Polychromophilus* isolated from *Eptesicus diminutus*) was grouped with all *P. murinus* sequences in a sister clade. The latter, likely *P. murinus,* presented 1% divergence in the *cytb* sequence compared to the other Brazilian or Panamanian sequences, and was obtained from a different genus of bats. Thus, the possibility of most Brazilian sequences being a different *Polychromophilus* species must be investigated.

The present study provides the first molecular description of *Polychromophilus* parasites in *Myotis ruber*, *Myotis riparius*, and *Eptesicus diminutus* from Brazil and confirms the presence of this parasite 50 years after its first and only report in Brazilian territory. Moreover, our results suggest the occurrence of two distinct *Polychromophilus* species infecting two different genera of hosts, improving the current knowledge on blood parasites infecting Brazilian bats. However, it is crucial to add additional molecular markers to the phylogenetic analysis for an in-depth investigation. A three-genome phylogenetic analysis for robust haemosporidian phylogenies has been recommended [53] and must be properly included as part of a follow-up paper. Moreover, additional studies including morphological observations of these parasites combined with molecular data are needed to resolve its taxonomy. Furthermore, due to the great Brazilian extensions and the immense diversity of species and biomes, new bat populations should be investigated to provide a complete portrait of the biology of host–parasite interactions.

## Figures and Tables

**Table 1:** Nucleic acid polymorphism in mitochondrial cytochrome b gene (*cytb*) sequences of *Polychromophilus* sp. isolates from Brazil (116, 198, 335, 650, and 69642) and Panama (MYOPA01).

Isolate	219	247	261	273	339	405	512	789	792	810	811	853	885	945	1086
116	C	T	A	T	T	T	T	T	C	C	T	C	A	T	A
198	C	T	A	T	G	T	T	T	T	C	T	C	A	T	G
335	C	T	A	T	G	T	T	T	T	C	T	C	A	T	G
650	T	T	C	A	A	C	T	C	T	T	C	T	T	T	A
69642	C	T	A	T	T	T	T	T	C	C	T	C	A	C	A
MYOPA01	C	C	A	T	T	T	G								

MYOPA01 has 595 bp and thus, there was no overlap for the nucleotides from 789–1086 with the Brazilian sequences (1116 bp). Gray columns show two nucleic acid substitutions found only in this isolate.

**Table 2:** Similarity percentage between the mitochondrial cytochrome b gene (*cytb*) sequences of *Polychromophilus* sp. found in different bats from Brazil and Panama (MYOPA01).

	Bat Species	116	198	335	650	69642	MYOPA01
116	*Myotis ruber*	1116	99%	99%	99%	99%	99%
198	*Myotis riparius*	1113	1116	100%	99%	99%	99%
335	*Myotis riparius*	1113	1116	1116	99%	99%	99%
650	*Eptesicus diminutus*	1105	1105	1105	1116	98%	98%
69642	*Myotis riparius*	1115	1112	1112	1104	1116	99%
MYOPA01	*Myotis nigricans*	592	591	591	587	592	595

**Table 3:** Occurrence of *Polychromophilus* sp. in this study and previous studies worldwide.

Country or Continent	Analyzed Samples	Positive Samples (Positivity)	Positive Host Species	Reference
Africa ^1^	505	56 (11%)	*Miniopterus africanus*, *M. fraterculus*, *M. minor*, *M. natalensis*, *M. rufus*, *Myotis tricolor*	[17]
Australia ^2^	85	47 (55%)	*Miniopterus orianae*	[32]
Brazil ^3^	406	5 (1.2%)	*Eptesicus diminutus*, *Myotis ruber*, *Myotis riparius*	This study
Europe ^4^	310	231 (74.5%)	*Miniopterus schreibersii*	[33]
Gabon	164	5 (3%)	*Miniopterus inflatus*	[34]
Gabon	92	2 (2%)	*Miniopterus minor*	[35]
Guinea	274	5 (2%)	*Miniopterus villiersi*, *Neoromicia capensis*, *Pipistrellus aff. grandidieri*	[15]
Madagascar	947	130 (13.5%)	*Paratriaenops furculus*, *Miniopterus aelleni*, *M. manavi*, *M. gleni, M. grifthsi*, *M. griveaudi*, *M. mahafaliensis*, *M. majori*, *M. sororculus*, *Myotis goudoti*	[36]
Madagascar	222	27 (12.2%)	*Miniopterus egeri*, *M. griveaudi*, *M. ambohitrensis*, *M. gleni*, *Scotophilus robustus*, *Myotis goudoti*	[37]
Switzerland	207	70 (34%)	*Myotis daubentonii*, *M. myotis*, *Nyctalus noctula*, *Eptesicus serotinus*	[38]
Thailand	44	5 (11%)	*Scotophilus kuhlii*	[39]
Thailand	271	13 (4.8%)	*Myotis siligorensis*, *Taphozous melanopogon*	[40]

^1^ Kenya, Malawi, Mozambique, Tanzania, and Uganda. ^2^ Detection of haemosporidians was performed by microscopy in all samples (274), but the molecular analysis was performed on only part of them (85 samples). ^3^ Detection of *Polychromophilus* was performed in samples of brain tissue. ^4^ Croatia, Portugal, Spain, Switzerland, Italy, Slovakia, and France.

## Data Availability

The data presented in this study are available in Appendix A and also in the GenBank^®^ database (https://www.ncbi.nlm.nih.gov/genbank/ (accessed on 19 March 2021)) (accession numbers MW984518-MW984522).

## Appendix A

**Table A1:** Mitochondrial cytochrome b gene (*cytb*) sequences of the parasite used as references for phylogenetic analyses and their respective accession numbers in the Genbank^®^ database.

GenBank^®^ Accession Number	Parasite Species	Host
HQ173882	*Eimeria magna*	Rabbit
HQ173892	*Eimeria vejdovskyi*	Rabbit
AY099045	*Haemoproteus majoris*	Bird
HM222472	*Haemoproteus* sp.	Bird
KT367832, KT367833, KT367822, KT367828	Haemosporida sp.	Antelope
KT367830, KT367819, KT367837	Haemosporida sp.	Antelope
FJ168565	*Hepatocystis* sp.	Bat
JQ070951, JQ070956	*Hepatocystis* sp.	Monkey
AY099063	*Leucocytozoon dubreuli*	Bird
NC_012450, FJ168563	*Leucocytozoon majoris*	Bird
KF159690, KF159720, MK098843-MK098847	*Nycteria* sp.	Bat
GQ141581, GQ141585, KT367845, KM598212	*Parahaemoproteus* sp.	Bird
NC_012447, FJ168561	*Parahaemoproteus vireonis*	Bird
HM235081	*Plasmodium adleri*	Gorilla
AY099054, HQ712051	*Plasmodium atheruri*	Rodent
AY099055	*Plasmodium azurophilum*	Lizard
KP875474	*Plasmodium billcollinsi*	Chimpanzee
HM235065	*Plasmodium blacklocki*	Gorilla
KF159674	*Plasmodium cyclopsi*	Bat
AB444126	*Plasmodium cynomolgi*	Monkey
FJ895307	*Plasmodium gaboni*	Chimpanzee
AF069612	*Plasmodium gallinaceum*	Bird
AY099053	*Plasmodium giganteum*	Lizard
JF923751	*Plasmodium gonderi*	Mandrill
JQ345504	*Plasmodium knowlesi*	Human
HM000110	*Plasmodium malariae*	Chimpanzee
GU723548	*Plasmodium ovale*	Human
JF923762	*Plasmodium praefalciparum*	Monkey
KP875479	*Plasmodium reichenowi*	Chimpanzee
AY733090	*Plasmodium relictum*	Bird
HM222485	*Plasmodium* sp.	Bird
JF923753	*Plasmodium* sp.	Mandrill
KJ700853, KJ700854	*Plasmodium vinckei*	Rodent
KF591834	*Plasmodium vivax*	Human
KF159671	*Plasmodium voltaicum*	Bat
DQ414658	*Plasmodium yoelii killicki*	Rodent

**Table A2:** Genbank^®^ accession numbers of *Polychromophilus* mitochondrial cytochrome b gene (*cytb*) sequences used as a reference for phylogenetic analyses and sequences found in this study.

GenBank Accession Number	Parasite Species	Host	Origin
KU318045	*P. melanipherus*	*Anopheles marshallii*	Gabon
HM055583	*P. murinus*	*Myotis daubentonii*	Switzerland
HM055583	*P. murinus*	*Eptesicus serotinus*	Switzerland
HM055583	*P. murinus*	*Nyctalus noctula*	Switzerland
HM055583	*P. murinus*	*Myotis myotis*	Switzerland
HM055584–HM055589	*P. murinus*	*Myotis daubentonii*	Switzerland
MW984521	*Polychromophilus* sp.	*Eptesicus diminutus*	Brazil (this study)
KT750375	*Polychromophilus* sp.	*Miniopterus africanus*	Kenya
MH744509–MH744511, MH744518, MH744521	*P. melanipherus*	*Miniopterus gleni*	Madagascar
MH744506, MH744519	*P. melanipherus*	*Miniopterus griffithsi*	Madagascar
MH744514–MH744516	*P. melanipherus*	*Miniopterus griveaudi*	Madagascar
MH744508, MH744522–MH744525	*P. melanipherus*	*Miniopterus griveaudi*	Madagascar
JQ995284–JQ995288	*Polychromophilus* sp.	*Miniopterus inflatus*	Gabon
MH744504, MH744505	*P. melanipherus*	*Miniopterus mahafaliensis*	Madagascar
MH744512, MH744526	*P. melanipherus*	*Miniopterus manavi*	Madagascar
KT750430	*Polychromophilus* sp.	*Miniopterus minor*	Tanzania
MK098848, MK098849	*Polychromophilus* sp.	*Miniopterus minor*	Gabon
MW007677	*P. melanipherus*	*Miniopterus natalensis*	South Africa
KT750376-KT750382, KT750401, KT750402	*Polychromophilus* sp.	*Miniopterus natalensis*	Kenya
KT750406, KT750408, KT750409	*Polychromophilus* sp.	*Miniopterus natalensis*	Kenya
MK088162–MK088168	*P. melanipherus*	*Miniopterus orianae*	Australia
KT750383-KT750386, KT750415, KT750418	*Polychromophilus* sp.	*Miniopterus rufus*	Kenya
JN990708–JN990711	*P. melanipherus*	*Miniopterus schreibersii*	Switzerland
KJ131270–KJ131277	*P. melanipherus*	*Miniopterus schreibersii*	Southern and Central Europe
MW007689	*P. melanipherus*	*Miniopterus schreibersii*	Spain
KT750389	*Polychromophilus* sp.	*Miniopterus* sp.	Tanzania
KT750387	*Polychromophilus* sp.	*Miniopterus* sp.	Kenya
KF159675, KF159681, KF159699	*Polychromophilus* sp.	*Miniopterus villiersi*	Guinea
JN990712, JN990713	*P. murinus*	*Myotis daubentonii*	Switzerland
MH744532–MH744536	*P. murinus*	*Myotis goudoti*	Madagascar
LN483038	*Polychromophilus* sp.	*Myotis nigricans*	Panamá
MW984519, MW984520, MW984522	*Polychromophilus* sp.	*Myotis riparius*	Brazil (this study)
MW984518	*Polychromophilus* sp.	*Myotis ruber*	Brazil (this study)
MT136168	*P. murinus*	*Myotis siligorensis*	Thailand
KF159700	*Polychromophilus* sp.	*Neoromicia capensis*	Guinea
MW007685	*P. melanipherus*	*Nycteribia schmidlii*	Spain
MW007680, MW007681	*P. melanipherus*	*Nycteribia schmidlii*	Hungary
MW007682	*P. melanipherus*	*Nycteribia schmidlii*	Italy
MW007671–MW007674, MW007676	*P. melanipherus*	*Nycteribia schmidlii scotti*	South Africa
KU182361–KU182367	*P. melanipherus*	*Nycteribia schmidlii scotti*	Gabon
MH744527	*P. melanipherus*	*Nycteribia stylidiopsis*	Madagascar
MH744520	*P. melanipherus*	*Paratriaenops furculus*	Madagascar
KU182368	*P. melanipherus*	*Penicillidia fulvida*	Gabon
MH744528–MH744531	*P. melanipherus*	*Penicillidia leptothrinax*	Madagascar
MH744537	*P. murinus*	*Penicillidia* sp.	Madagascar
KF159714	*Polychromophilus* sp.	*Pipistrellus aff. grandidieri*	Guinea
LN483036	*P. murinus*	*Rhinolophus* sp.	Bulgaria
MT750305–MT750309	*Polychromophilus* sp.	*Scotophilus kuhlii*	Thailand
MT136167	*P. melanipherus*	*Taphozous melanopogon*	Thailand
